# Formation and Properties of Electrospun Zein Nanofibers from Glacial Acetic Acid Solutions: Effects of Process Variables and Surfactants

**DOI:** 10.3390/foods15173156

**Published:** 2026-09-05

**Authors:** Shumin Wang, Xiaolin Liang, Hanyu Chai, Shaohua Lin, Yankun Fu, Xiaofei Liu, Hongyu Li, Pengjie Wang

**Affiliations:** 1 College of Food and Bio-Engineering, Beijing Vocational College of Agriculture, Beijing 104224, China; sdutshumin@163.com (S.W.); 86001@bvca.edu.cn (S.L.); liuxiaofei0054@163.com (X.L.); 2 Department of Nutrition and Health, China Agricultural University, Beijing 100193, China; lynnloeng13@163.com (X.L.); b20253311554@cau.edu.cn (H.C.); hntyfyk2021@163.com (Y.F.); 3 Liaocheng Academy of Agricultural Sciences, Liaocheng 252000, China

**Keywords:** zein nanofibers, electrospinning, surfactants, diameter modulation

## Abstract

Electrospun zein nanofibers are promising bio-based materials, but producing uniform ultrafine fibers with desirable performance remains challenging. This study examined the effects of process variables and three surfactants on zein fiber formation, diameter, and material properties. Zein solutions (30–40%, *w*/*v*) were prepared in glacial acetic acid, and 2% (*w*/*w*, based on zein) triethyl benzyl ammonium chloride (TEBAC), sodium dodecyl sulfate (SDS), or span-80 was incorporated into the 30% zein solution. Zein concentration and feed rate yielded fiber diameters of 208.22–1001.90 nm. Surfactants reduced surface tension and increased conductivity, promoting jet stretching and generating uniform fibers with diameters near 100 nm. SDS produced the smallest fibers (98.92 ± 15.20 nm). TEBAC increased tensile strength from 11.08 to 63.26 MPa and improved dimensional retention in water, whereas SDS and span-80 increased elongation at break but reduced strength and stiffness. All surfactants increased wettability and altered intermolecular interactions and secondary structure of zein, although they reduced thermal stability. Overall, surfactants acted as both electrospinning aids and structure-directing modifiers, with TEBAC providing the best balance of fineness, strength, and aqueous stability. These ultrafine, robust, and water-stable nanofibers are promising as functional coating layers for high-moisture food packaging and as carriers for antioxidants or antimicrobial agents.

## 1. Introduction

Electrospinning is a simple, cost-effective, and continuous technique for producing fibers with diameters spanning the nanoscale to the microscale. Electrospun fibrous materials generally possess a high specific surface area and an interconnected porous structure, which can facilitate water vapor transmission in packaging and enable the efficient loading and controlled release of active compounds [1]. In addition, electrospinning is capable of fabricating nanofibers with different morphologies and sizes (diameters ranging from nanometers to micrometers) [2,3]. These advantages endow electrospinning technology with broad application prospects in active food packaging, drug delivery, sensing, filtration, and energy harvesting [4]. To further expand its application scope, it is essential to precisely control the morphology and size of electrospun nanofibers.

The morphology and diameter of electrospun fibers can be adjusted by various parameters, including the electrospinning process conditions (e.g., voltage, collection distance, flow rate, nozzle diameter), environmental factors (e.g., temperature, humidity), and the characteristics of the polymers and their solutions (e.g., molecular weight, concentration, viscosity, conductivity, surface tension, polarity) [5,6,7,8]. Typically, lower solution conductivity promotes the production of fibers with larger diameters by reducing the jet elongation rate along the fiber axis [8]. Fiber diameter decreases as the applied voltage increases due to the enhanced electrostatic stress on the jet [9]. Because more solution is delivered per unit time, a higher flow rate usually leads to an increase in fiber diameter. The collection distance affects fiber morphology by altering the electric field strength [10]. Furthermore, the polymer concentration has a significant effect on the diameter of electrospun fibers, and a decrease in polymer concentration leads to a reduction in fiber diameter. However, according to previous reports, it remains difficult to obtain electrospun fibers with a diameter of less than 200 nm. The main reason is that the excessive dilution of the polymer solution destabilizes the Taylor cone, resulting in beaded fibers or nanoparticles.

The addition of surfactants can stabilize the jet, thereby helping to produce uniform electrospun fibers with small diameters and narrow diameter distributions [11,12,13]. On the one hand, when their concentrations exceed their respective critical micelle concentrations, surfactants can self-assemble to form micelles, which can improve the solubility of active compounds and control their release behavior. On the other hand, surfactant molecules contain hydrophobic and hydrophilic groups, which can spontaneously adsorb at the oil–water interface and reduce the surface tension of the solution. Meanwhile, interactions between polymers and surfactants can also alter the viscosity and conductivity of the solution, thereby affecting the electrospinning process [13]. Almeida et al. (2020) prepared cellulose acetate nanofibers with smaller diameters and fewer bead defects after adding the cationic surfactant cetylpyridinium bromide [11]. In the study by Beigmoradi et al. (2021), the addition of the anionic surfactant sodium dodecyl sulfate and the cationic surfactant cetyltrimethylammonium bromide not only reduced the diameter of electrospun polysulfone fibers, but also modified the surface roughness of the nanofibers [14]. Therefore, surfactant–assisted electrospinning may be an effective means to produce fibers with smaller diameters (<200 nm).

Both natural and synthetic polymers can be used to produce electrospun nanofibers. From the perspectives of food safety and environmental sustainability, the development of electrospun nanofibers from biopolymers is of great significance. As a by-product of the bioethanol and starch industries, zein possesses biodegradability, biocompatibility, thermal resistance, and antioxidant properties, supporting its potential use in food packaging and bioactive-delivery materials. For example, electrospun zein fibers have been developed as antimicrobial packaging matrices to inhibit foodborne pathogens and extend food shelf life, and as delivery systems to improve the stability and controlled release of bioactive compounds such as curcumin [15,16]. Its solubility in suitable volatile solvents and its ability to form sufficiently entangled polymer solutions enable zein to be processed into continuous fibers by electrospinning [17,18]. In addition, zein is one of the few proteins that can be electrospun using food-safe solvents, and glacial acetic acid has been proven to be a good solvent for zein. Among the available solvent systems, glacial acetic acid is particularly attractive from both food-safety and processing perspectives. It is regarded as a food-safe solvent for food-related electrospinning, while also enabling more continuous electrospinning and fibers with a rounder morphology and narrower diameter distribution than conventional aqueous alcohol systems [19,20]. In recent years, the physicochemical properties and functionality of electrospun zein nanofibers have been extensively studied, particularly in relation to their use as nanomaterials for the protection, delivery, and sustained release of active substances [21,22,23,24]. However, there is relatively little research on the systematic regulation of the size of electrospun fibers from biopolymers and on surfactant–assisted biopolymer nanofibers [25,26,27,28]. In addition, no definitive conclusion has been reached regarding the influence of different surfactants on the formation of the electrospun nanofibers and the underlying mechanisms governing fiber size.

In this study, electrospun nanofibers were prepared using zein as the biopolymer and glacial acetic acid as the green solvent. The size of the fibers was regulated by adjusting electrospinning process conditions and adding surfactants. The morphology, mechanical properties, surface wettability, water stability, thermal behavior, and molecular structure of zein nanofibers with different sizes were analyzed. The effects of an anionic surfactant (sodium dodecyl sulfate), a nonionic surfactant (span-80), and a cationic surfactant (triethyl benzyl ammonium chloride) on the spinnability of zein were investigated. Additionally, the corresponding formation mechanism of zein nanofibers was explored. This study provides a theoretical foundation for the development and design of effective electrospun nanofibers from biopolymers, and supports the further expansion of their applications in various fields.

## 2. Materials and Methods

### 2.1. Materials

Zein was purchased from Sigma–Aldrich (St. Louis, MO, USA). Glacial acetic acid (100%) was purchased from Aladdin Inc. (Shanghai, China). Sodium dodecyl sulfate (SDS), span-80, and triethyl benzyl ammonium chloride (TEBAC) were purchased from Shanghai Macklin Biochemical Co., Ltd. (Shanghai, China).

### 2.2. Preparation of Electrospun Zein Nanofibers

Different amounts of zein powder were added to glacial acetic acid and fully dissolved by magnetic stirring (MYP11-2, Shanghai Meiyingpu Instrument Manufacturing Co., Ltd., Shanghai, China) overnight at room temperature (25 ± 2 °C) to prepare zein solutions with different concentrations of 30%, 35%, and 40% (*w*/*v*). The resulting zein solutions were allowed to stand for 10 min to remove air bubbles before electrospinning. For each experiment, a fixed volume of 2 mL of the feed solution was loaded into a 10 mL syringe and electrospun using an electrospinning apparatus (HZ-11, Qingdao Nuokang Environmental Protection Co., Ltd., Qingdao, China). The same feed-solution volume was used for all samples to improve the comparability of the resulting nanofiber mats. The electrospinning parameters were set as shown in Table 1. By varying the mass concentration (30–40%, *w*/*v*) of zein and flow rate (0.5–3 mL/h), four nanofiber samples were prepared and initially designated according to their processing conditions as ZNF_30_0.5, ZNF_35_1, ZNF_40_1, and ZNF_40_3, respectively. Their average fiber diameters were subsequently determined by SEM analysis.

A 30% (*w*/*v*) zein solution was dissolved in glacial acetic acid, and the dispersion was magnetically stirred overnight at room temperature (25 ± 2 °C) to ensure complete dissolution. Then, the surfactants were separately added to the zein solution at a concentration of 2% (*w*/*w*, based on zein). After continuous stirring for 2 h at room temperature, the resulting solutions were used for electrospinning. Under the electrospinning conditions listed in Table 1, zein nanofibers prepared with the cationic surfactant (TEBAC), anionic surfactant (SDS), and nonionic surfactant (span-80) were prepared and designated as TEBAC/ZNF, SDS/ZNF, and span-80/ZNF, respectively.

### 2.3. Characterization of the Feed Solution

The electrical conductivity was measured using a digital conductivity meter (Starter 300, OHAUS Corporation, Parsippany, NJ, USA). The viscosity was determined with a rotational rheometer (DHR, TA Instruments, New Castle, DE, USA), and the viscosity was recorded at a shear rate of 100 s^−1^. The surface tension was measured using a surface tensiometer (ADVance, KRÜSS GmbH, Hamburg, Germany). All tests were conducted in triplicate for each feed solution at room temperature (25 ± 1 °C).

### 2.4. Morphology and Diameter Distribution of Nanofibers

The morphology of zein nanofibers was observed using a scanning electron microscope (Hitachi, Tokyo, Japan). Before imaging, nanofiber samples were sputter-coated with gold. The diameter distribution was analyzed using ImageJ software (version 1.8.0), and 200 randomly selected fibers were measured for each sample.

### 2.5. Mechanical Properties

The tensile testing of zein nanofibers was performed using a universal testing machine (Instron, Norwood, MA, USA) according to a previously reported method [29]. Briefly, the nanofiber samples were cut into a size of 30 mm × 3 mm (length × width). The tensile properties were then determined at a crosshead speed of 5 mm/min and a gauge length of 5 mm. Five specimens from each nanofiber sample were tested. The tensile strength (TS), elongation at break (EB), and Young’s modulus (YM) were calculated according to the following equations:

(1) $$TS=\frac{F_{m}}{S}$$

(2) $$EB=\frac{\left(L_{b}\times L_{0}\right)}{L_{0}}\times 100\%$$

(3) $$YM=\frac{\left(F_{m}\times L_{g}\right)}{\left(S\times L_{m}\right)}$$

where *F_m_* is the maximum recorded load (N); *S* is the cross–sectional area of the nanofiber samples (mm^2^); *L_b_* is the specimen length at break of nanofiber samples (mm); *L*_0_ is the initial length of nanofiber samples (mm); *L_g_* is the gauge length (mm); *L_m_* is the specimen length at the maximum load (mm).

### 2.6. Water Contact Angles (WCAs)

The WCAs of the zein nanofiber mats were measured using a contact angle meter (OCA 25, DataPhysics Instruments GmbH, Filderstadt, Germany). The nanofiber mats were cut into specimens of the same size and attached flat onto glass slides. A 3 μL droplet of distilled water was then deposited onto the surface of each mat. Images of the water droplets were recorded within 5 s, and the WCA values were calculated from the recorded images. Five independent measurements were performed for each sample. The thickness and density of the nanofiber mats were not independently determined in this study; however, the same volume of feed solution was used for the preparation of each sample to improve comparability among the resulting mats.

### 2.7. Immersion Study

An immersion test was performed on zein nanofiber samples to evaluate their stability in water. According to our previous reports, each nanofiber sample was cut into a size of 2.5 cm × 2.5 cm and then immersed in 35 mL of deionized water for 24 h [17,29]. After immersion, the tested samples were freeze-dried, and their morphology was examined using scanning electron microscopy (SEM). In addition, macroscopic images of the nanofiber samples before and after immersion in water were captured.

### 2.8. Thermal Properties

Differential scanning calorimetry (DSC) measurements were performed following the method described by Maria Leena et al. (2020) [30], on a TA instruments Q2000 DSC (TA Instruments, New Castle, DE, USA). Each sample (5.0 ± 0.1 mg) was accurately weighed into an aluminum pan and hermetically sealed. The samples were first heated from 25 to 200 °C at a rate of 10 °C/min, subsequently cooled from 200 to −10 °C at 10 °C/min, and then reheated from −10 to 200 °C at the same rate. All measurements were conducted under a nitrogen atmosphere. The first heating scan was used to eliminate the previous thermal history of the samples, and the glass transition temperature (T_g_) was obtained by analyzing the second heating scan using TRIOS software (version 4.5, TA Instruments, New Castle, DE, USA; https://www.tainstruments.com/support/software-downloads-support/downloads/ (accessed on 31 August 2026)).

Thermogravimetric analysis (TGA) was conducted using an STA 449 F5 instrument (Netzsch Gerätebau GmbH, Selb, Germany) as described previously. Approximately 5 mg of each nanofiber sample was placed in a suitable sample pan and heated continuously from 25 to 600 °C at a rate of 10 °C/min. The changes in sample mass as a function of temperature were recorded to evaluate the thermal degradation behavior and thermal stability of the nanofibers.

### 2.9. Attenuated Total Reflectance Infrared Spectroscopy (ATR–FTIR)

Following the method described by Isik et al. (2025) [31], the functional groups and intermolecular interactions in zein nanofiber samples were investigated via a Fourier transform infrared (FTIR) spectrometer (Nicolet 6700, Thermo Scientific, Waltham, MA, USA) equipped with an attenuated total reflection (ATR). The spectra were collected in the range of 4000–400 cm^−1^ and at a resolution of 4 cm^−1^. A total of 32 scans were collected for each sample. The secondary structure in the amide I region (1600–1700 cm^−1^) was evaluated using PeakFit software (version 4.12, Seasolve Software, Framingham, MA, USA) and OMNIC 9.2 software (Thermo Fisher Scientific Inc., Carlsbad, CA, USA) [17].

### 2.10. Statistical Analysis

Statistical analyses were performed using SPSS Statistics software (version 20.0, IBM Corp., Armonk, NY, USA). The data are expressed as the mean ± standard deviation (*n* = 3). One-way analysis of variance (ANOVA) and Duncan’s multiple range test were used to identify statistically significant differences among treatments (*p* < 0.05).

## 3. Results

### 3.1. Physical Properties of Spinning Solutions

#### 3.1.1. Properties of Zein Solutions with Different Concentrations

The physical properties of the spinning solutions strongly influence the diameter and morphology of electrospun fibers. The viscosity, conductivity, and surface tension of the solutions with different zein concentrations or different surfactants are presented in Table 2. In Table 2, the samples are identified using process-based labels according to the zein concentration and flow rate employed during electrospinning, because the fiber diameters were determined only after SEM characterization. Specifically, ZNF_30_0.5, ZNF_35_1, ZNF_40_1, and ZNF_40_3 exhibited average fiber diameters of 208.22 ± 46.93, 391.40 ± 60.85, 602.28 ± 82.52, and 1001.90 ± 121.33 nm, respectively. For convenience, these samples are subsequently referred to as ZNF_200, ZNF_400, ZNF_600, and ZNF_1000 in the discussion and figures. Among these feed solutions, the 30% (*w*/*v*) zein solution used to prepare ZNF_200 exhibited a viscosity of 0.32 Pa·s, a conductivity of 103.73 μS/cm, and a surface tension of 46.10 mN/m. As the zein concentration increased to 40%, the viscosity of the spinning solution (ZNF_600 and ZNF_1000) significantly rose (1.06 Pa·s), and the surface tension also increased (47.98 mN/m). Meanwhile, the conductivity exhibited an initial increase followed by a decrease, with the 35% zein solution (ZNF_400) showing the highest conductivity (113.67 μS/cm). Guo et al. [32] confirmed that the viscosity of spinning solutions rises with increasing polymer concentration. Coelho et al. [10] similarly reported the viscosity and conductivity of zein solutions were also found to increase with higher zein concentrations; while the inconsistent changes in conductivity may be attributed to the use of different solvents in the spinning solution.

#### 3.1.2. Properties of Zein Solutions Added with Surfactants

At a dosage of 2%, all three surfactants significantly decreased the surface tension and increased the conductivity of the spinning solution (Table 2). Among them, the addition of the cationic surfactant TEBAC resulted in the highest conductivity (225.67 μS/cm) and the lowest surface tension (36.72 mN/m) of zein solution. Additionally, the addition of cationic surfactant TEBAC and non-ionic surfactant (span-80) caused only minor changes in viscosity, whereas the addition of the anionic surfactant SDS significantly increased the viscosity. These results indicate that there is an interaction between zein and surfactants. Interactions among polymer chains, surfactants, and solvent molecules may lead to the formation of polymer–surfactant complexes in solution, which can greatly alter the properties of polymer solutions [33]. The surfactant molecules form micelles in the solution when their concentration exceeds the critical micelle concentration. The micelle aggregates may associate with zein chains or adsorb onto the surfaces of zein aggregates, thereby reducing the surface tension of zein solution and improving the electrospinning performance of zein. Moreover, because of their mobility within the polymer jet, surfactant molecules may migrate toward the air–liquid interface and alter the surface charge density. Consistent with the findings of Wang et al. [12], the presence of the nonionic surfactant Triton X–100 was found to decrease the surface tension and enhance the electrospinnability of polyvinylpyrrolidone solutions.

The viscosity of a polymer solution is a key factor affecting its spinnability and fiber morphology. Solution viscosity is influenced by polymer concentration, molecular weight, molecular structure, and solvent type [34]. As shown in Table 2, the addition of 2% (*w*/*w*, based on zein) SDS and TEBAC increased the viscosity from 0.32 Pa·s to 0.38 Pa·s and 0.34 Pa·s; and as the concentrations increased, the viscosity of their mixed solutions presented a trend of first decreasing and then increasing, but their viscosities are still higher than that of the pure zein solution. The addition of 2% (*w*/*w*, based on zein) span-80 caused little change in viscosity of zein solution (0.34 Pa·s); when the concentration was increased to 10% (*w*/*w*, based on zein), the viscosity of the mixed solution significantly decreased; as the concentration continued to increase to 20% (*w*/*w*, based on zein), the viscosity of the zein/span-80 mixed solution increased. After the addition of SDS, the positively charged zein molecules can interact electrostatically with the negatively charged head groups of SDS. This interaction impedes solvent evaporation, thereby leading to an increase in the viscosity of the solution. The added TEBAC may interact with the hydrophobic groups of zein molecules, leading to enhanced binding between zein molecular chains and increasing viscosity. Span-80 does not introduce additional ionic species, and its interaction with zein is likely dominated by non-electrostatic forces. Consequently, with the increase in surfactant concentration, the viscosity of SDS/zein and TEBAC/zein solutions both increase, while the viscosity of the span-80/zein mixed solution remains unchanged or slightly decreases. Jia et al. [35] investigated the interactions between cationic, anionic, zwitterionic, and nonionic surfactants and polyvinyl alcohol, as well as their effects on the conductivity, surface tension, and viscosity of the solution, and obtained similar results.

The surface tension of a solution is generally defined as the force acting perpendicular to any line of unit length on the liquid surface [36]. During electrospinning, jet formation begins when the electrostatic repulsion within the solution overcomes the surface tension of the solution. In general, a lower surface tension of the spinning solution facilitates electrospinning at a relatively lower electric field strength. In all cases, the zein/surfactant mixtures exhibited lower surface tensions than the pure zein solutions. Among the three surfactants, SDS and TEBAC significantly reduced the surface tension of the zein solutions. As shown in Table S1, the surface tension of the corresponding mixture solution of zein/surfactant gradually increased with the concentration of the three surfactants (TEBAC, SDS, and span-80) rising from 2% to 20%. This is regarded as evidence of the interaction between zein and the three surfactants, and these interactions varied as the surfactant concentration increased.

According to reports, when the content is below the critical micelle concentration (CMC), the surfactant exists in the form of single molecules and hardly interact with the polymer in the solution; when the concentration reaches the CMC, the surfactant molecules begin to interact with the polymer, and as the concentration increases, the surfactant forms free micelles; When the interaction between the polymer and the surfactant reaches saturation, a further increase in surfactant content will no longer affect the interaction between the polymer and the surfactant [35]. In this study, a surfactant dosage of 2% (*w*/*w*, based on zein) was used, which was considered to be higher than the CMC. In this case, interactions existed between zein and the surfactant, yet the saturation concentration was not reached. This was because the conductivity and surface tension of the spinning solution both changed with the increase in surfactant content; moreover, due to the excess free micelles, the surface tension increased as the surfactant concentration increased (Table S1).

According to Table S1, the conductivity of the zein solution was 103.73 µS/cm. After adding 2% (*w*/*w*, based on zein) of TEBAC, SDS and span-80 respectively, the conductivity of the zein/surfactant mixed solutions increased to 225.67, 154.37 and 120.23 µS/cm. As the concentration of the surfactant increased to 20%, the conductivity of the TEBAC/zein mixed solution gradually rose to 662.00 µS/cm. In contrast, the conductivity of the SDS/zein mixed solution exhibited a decrease–then–increase trend with the increase in SDS concentration, reaching a value of 200.83 µS/cm when the SDS concentration was 20%. However, the conductivity of the span-80/zein mixed solution decreased with the increase in span-80 concentration, and it was 63.73 µS/cm at a span-80 concentration of 20%. These results indicate that the physical properties of the surfactant/zein mixed solutions depend on the content and type of the surfactant.

### 3.2. Morphology and Diameter Distribution of Zein Nanofibers

**Figure 1:**
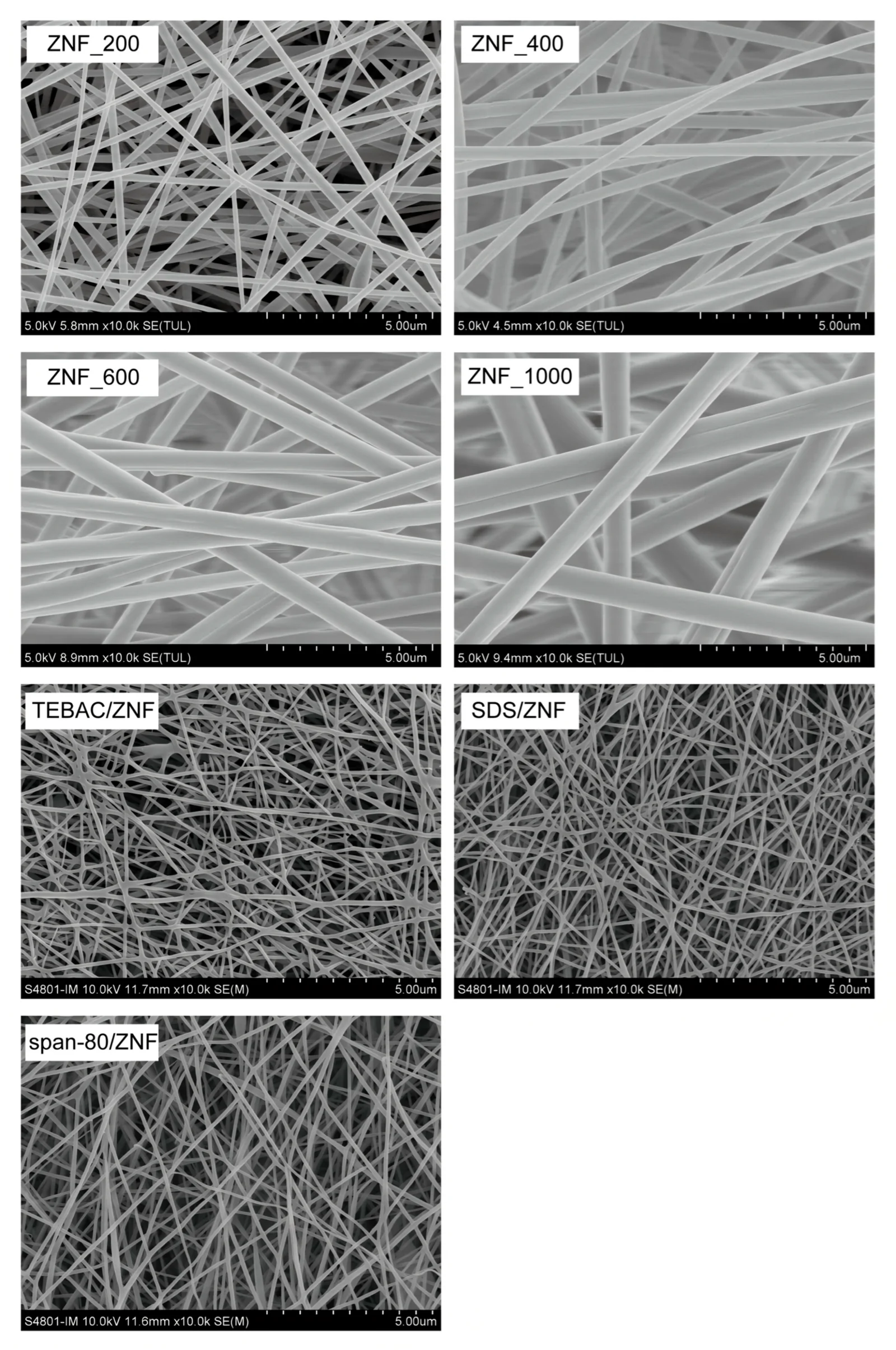
SEM images and size frequency distribution diagram of zein nanofibers with different diameters and with surfactants added.

The microscopic morphology of zein nanofibers with different diameters was observed by SEM (Figure 1). The results showed that the fiber diameter largely depends on the mass concentration of zein, and the diameter of the fiber increased with increasing of zein concentration (Table 2, Figure S2). The possible reason is that as the concentration of zein increases, the viscosity of the spinning solution increases, promoting the entanglement of zein molecular chains in the solution, ultimately resulting in an increase in fiber diameter. Previous studies have also indicated that polymer concentration is one of the most important parameters affecting fiber diameter [37]. Despite their different diameters, all the fibers exhibited a rod–like morphology with a uniform appearance; this indicates that changes in the mass concentration of zein and the flow rate can affect the fiber diameter, but they exert no influence on the fiber shape.

Compared with the sample without surfactants (ZNF_200), the addition of surfactants resulted in a more uniform fiber morphology, manifested by a narrower diameter distribution. In addition, the incorporation of each of the three surfactants into the solution significantly reduced the diameter of zein fibers; when added at a concentration of 2% (*w*/*w*, based on zein), the average diameters of TEBAC/ZNF, SDS/ZNF, and span-80/ZNF were 106.10 ± 18.58 nm, 98.92 ± 15.20 nm, and 106.11 ± 17.98 nm, respectively. Among these samples, SDS/ZNF exhibited the smallest average diameter and the narrowest diameter distribution. Previous studies have also reported similar results, in which surfactants such as Tween 80, SDS, CTAB, and phospholipids were added to reduce the fiber diameter of zein nanofibers [13]. In a study by Jia et al. [35], the addition of a surfactant to the polyvinyl alcohol solution resulted in fibers with smaller average diameters. On the one hand, this phenomenon might be attributed to the formation of polymer–surfactant complexes, which weakens the interactions between zein molecular chains [14]. On the other hand, the incorporation of the surfactant can alter the solution properties, such as reducing surface tension and increasing electrical conductivity, both of which facilitate the stretching of fibers and ultimately lead to a decrease in fiber diameter [38]. Sari et al. also found that higher charge density leads to enhanced electrostatic force, yielding electrospun fibers with smaller diameters [39]. In addition, the reduction in the surface tension of the spinning solution induced by the addition of surfactants also facilitates the fabrication of more uniform fibers, resulting in a narrower fiber diameter distribution. For TEBAC/ZNF and SDS/ZNF, the difference in their fiber diameters may be due to electrostatic interactions.

### 3.3. Mechanical Test

**Figure 2:**
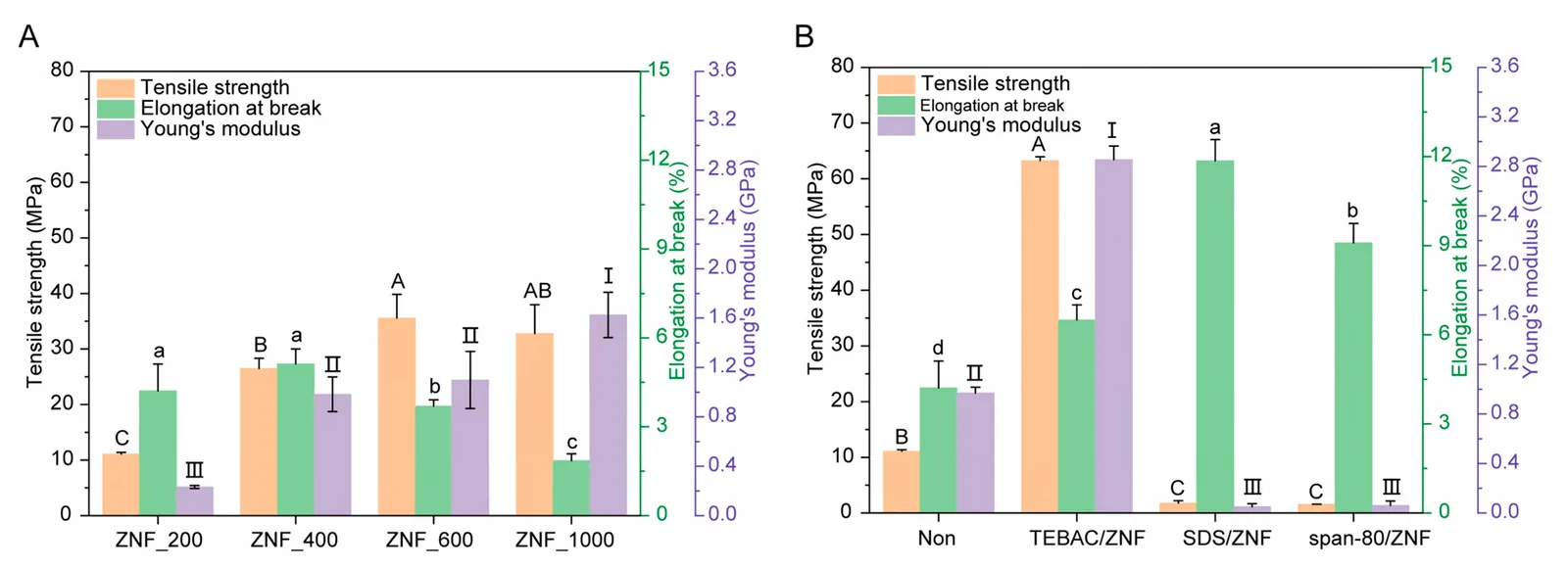
The mechanical properties of zein nanofibers with different diameters (**A**) and with surfactants added (**B**). Different uppercase letters, lowercase letters, and Roman numerals indicate significant differences in tensile strength, elongation at break, and Young’s modulus, respectively (*p* < 0.05).

The mechanical properties of zein nanofibers with different diameters and with surfactants added were shown in Figure 2. The results showed that the TS, EB, and YM values of ZNF_200 were 11.08 MPa, 4.21%, and 0.97 GPa, respectively. The TS of zein nanofiber samples showed a change of first increasing and then decreasing with the increase in fiber diameter; ZNF_600 had the maximum TS of 35.55 MPa. Similarly, the YM of zein nanofiber samples gradually increase from 0.97 GPa to 6.77 GPa with the increase in fiber diameter. However, the EB of zein nanofiber samples gradually decreased with the increase in fiber diameter; ZNF_1000 had the minimum EB value of 1.86%. Figure 2B presents the mechanical properties of the zein nanofibers and the zein nanofibers assisted by various surfactants. It can be seen that the mechanical properties of zein nanofibers are largely influenced by the type of surfactant. Compared with the nanofibers without surfactant (None), the TS, EB, and YM of TEBAC/ZNF increased to 63.26 MPa, 6.50%, and 2.86 MPa, respectively, indicating that the addition of the cationic surfactant TEBAC can significantly improve the mechanical properties of zein nanofibers. In the study conducted by de Almeida et al. (2020), the addition of cetylpyridinium bromide, a cationic surfactant, was also found to enhance the mechanical properties of cellulose acetate nanofibers [11]. In addition, the incorporation of the anionic surfactant SDS and the nonionic surfactant span-80 increased the EB of the zein nanofibers to 11.86% and 9.10%, respectively, while decreasing the TS and YM to 1.78 MPa, 1.55 MPa and 0.05 GPa, 0.06 GPa, respectively. In the study by Shan et al. [33], the incorporation of the non-ionic surfactant TX–15 was shown to improve the TS, EB, and elastic modulus of the chitosan/polylactic acid nanofibers. The inconsistency between their findings and those of the present study might be ascribed to the differences in the types of polymers and surfactants employed, which induce distinct interactions and consequently lead to different mechanical properties.

### 3.4. Surface Properties of Zein Nanofibers

**Figure 3:**
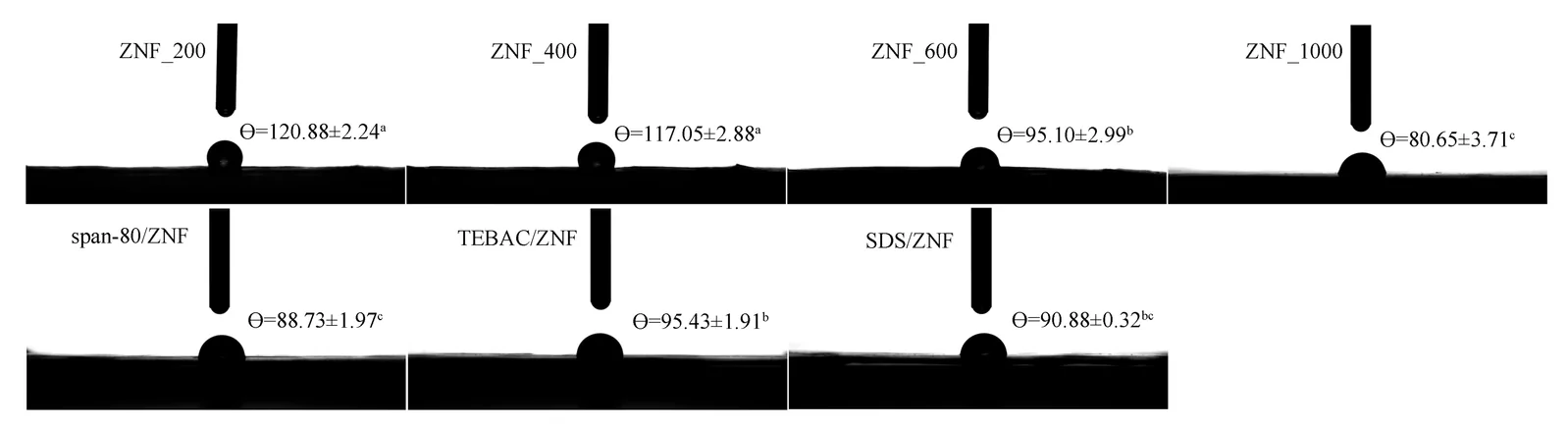
Images of water contact angle determinations for zein nanofiber mats with different diameters and surfactants added. Different lowercase letters indicate significant differences among samples (*p* < 0.05), whereas values sharing at least one letter are not significantly different.

Contact–angle measurement is a useful method for characterizing the surface wettability of materials. In this study, water was used as the measuring medium to determine the water contact angles of zein nanofibers with different diameters. The results in Figure 3 showed that as the fiber diameter increased from 200 nm to 1000 nm, the WCA value of the zein nanofibers decreased from 120.88° to 80.65°, indicating that the hydrophilicity of the nanofibers increased with the increase in fiber diameter. This may be due to the fact that the pores between fibers expand as the fiber diameter increased, which is more conducive to water penetration. In the study by Liu et al. [40], the WCA of PCL nanofibers was found to be independent of fiber diameter; the inconsistent results may be attributed to the use of different polymers, which consequently possess distinct surface properties. After adding the surfactants TEBAC, SDS and span-80, the WCA of the zein nanofibers decreased significantly. The WCA values of span-80/ZNF, TEBAC/ZNF, and SDS/ZNF were 88.73°, 95.43°, and 90.88°, respectively, indicating that the addition of surfactants increases the hydrophilicity of zein nanofibers. In the study conducted by Shan et al. [33], the presence of the non-ionic surfactant TX–15 in the polylactic acid/chitosan composite nanofibers resulted in an increase in the hydrophilicity of the composite nanofibers. Previous studies have indicated that surface roughness can significantly alter water contact–angle values as surface morphology varies [14]. The Wenzel model describes the contact angle on a real rough surface with chemical homogeneity, holding that an increase in surface roughness can enhance the hydrophilicity and hydrophobicity of hydrophilic and hydrophobic surfaces, respectively; in other words, surface roughness can amplify the wettability of the original surface [41]. As shown in Figure 3, the addition of span-80, TEBAC and SDS may increase the hydrophilicity of zein nanofibers by reducing their surface roughness. It has been reported that during the electrospinning process, a large number of surfactant molecules accumulate on the surface of nanofibers when they are formed and before they are solidified, which reduces the solvent evaporation rate and thus decreases the surface roughness of nanofibers [14]. In addition, the scanning electron microscopy images (Figure 1) show that the small–diameter fibers generated after the addition of surfactants help to increase the density of the zein nanofibers, which leads to an increase in the surface area of the fiber network, thereby enhancing the water permeability and hygroscopicity of the nanofiber matrix [33].

### 3.5. Water Stability of Zein Nanofibers

**Figure 4:**
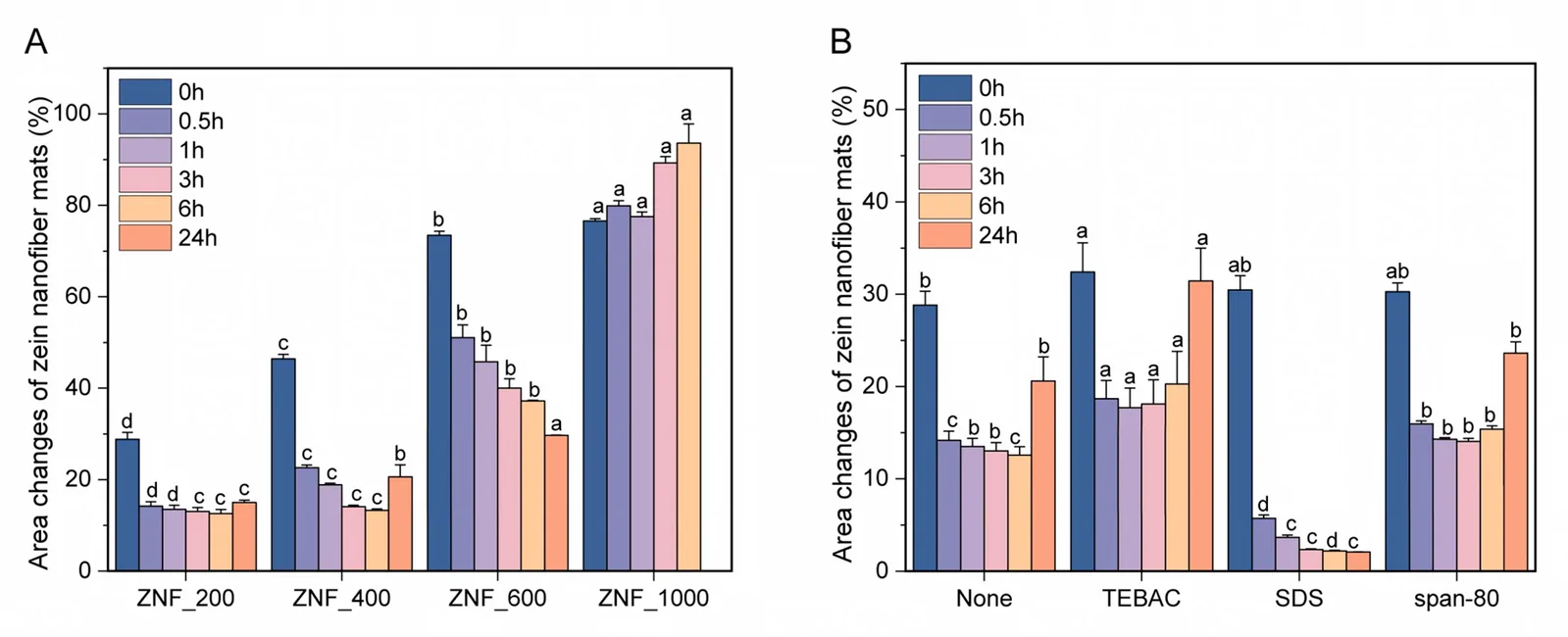
Area changes of zein nanofiber mats with different diameters (**A**) and with surfactants added (**B**) after immersion in water for 24 h. Different lowercase letters indicate significant differences among samples at the same immersion time (*p* < 0.05). Bars sharing the same letter are not significantly different.

The nanofiber mats were immersed in deionized water for 24 h to observe the macroscopic morphology (Figure S1) and area changes during water immersion (Figure 4). It was observed that upon initial contact with deionized water, all nanofiber samples exhibited shrinkage; the area of ZNF_200, ZNF_400, ZNF_600 and ZNF_1000 decreased to 28.83%, 46.41%, 73.45% and 76.58% of their initial areas, respectively. As the immersion time in deionized water increased, the areas of ZNF_200, ZNF_400, and ZNF_600 gradually decreased; after 24 h of immersion, their areas decreased to 20.60%, 15.00%, and 29.68% of their initial areas, respectively. However, ZNF_1000 swelled when exposed to deionized water. After immersion in deionized water for 6 h, the area of ZNF_1000 was 93.60% of its initial area; as the immersion time continued to increase, ZNF_1000 lost its integrity due to dissolution (Figure S1). These results indicate that the fiber diameter of zein mat nanofibers exerts a significant influence on their stability in aqueous media.

**Figure 5:**
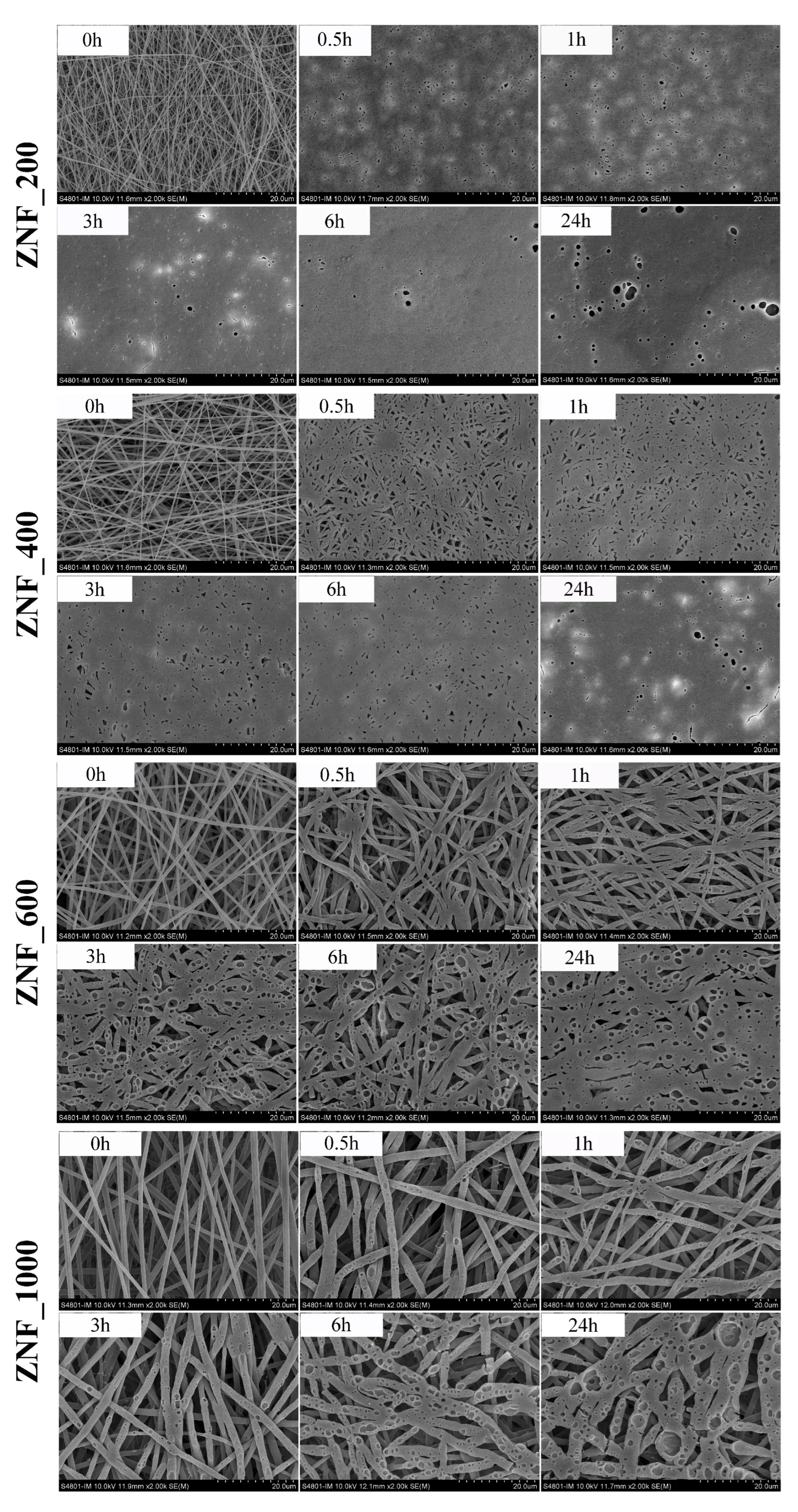
SEM images of zein nanofiber mats with different diameters.

**Figure 6:**
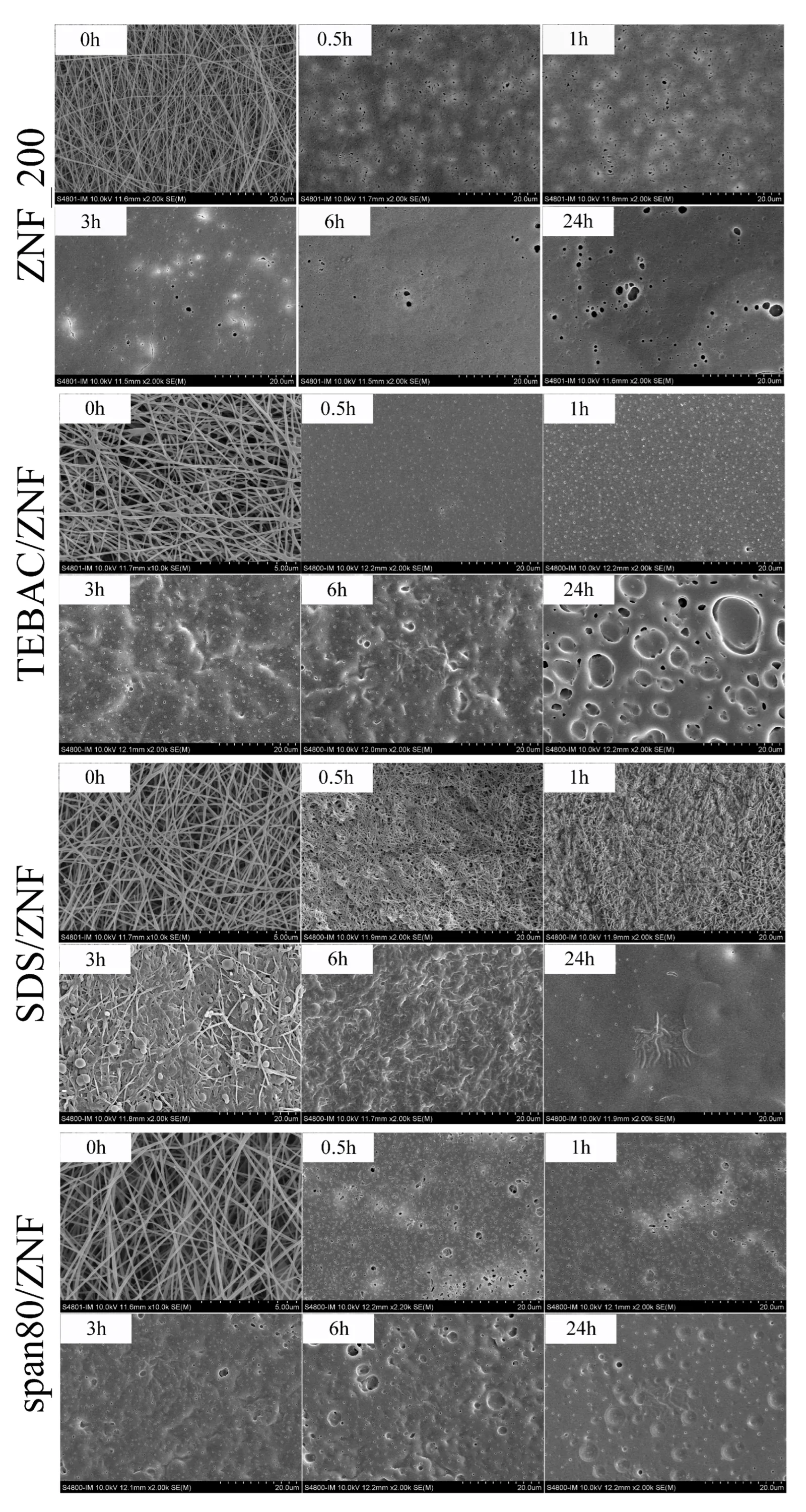
SEM images of zein nanofiber mats with surfactants added.

After the addition of surfactant, the swelling behavior of zein nanofibers in deionized water underwent significant changes. As shown in Figure 4, the presence of cationic surfactant TEBAC resulted in an increase in the area of the zein nanofiber sample from 28.83% to 32.41% of its initial volume. After immersion in deionized water for 24 h, the volume of TEBAC/ZNF reached 31.43% of its initial area, significantly higher than that of ZNF_200, which was 20.60%. Unlike cationic surfactants, the addition of anionic surfactant SDS led to more severe shrinkage of zein nanofibers when exposed to deionized water. After immersion in deionized water for 24 h, the volume of SDS/ZNF was only 2.07% of its initial value. In addition, the addition of nonionic surfactant span-80 had no significant effect on the swelling behavior of zein nanofibers in deionized water. After immersion in deionized water for 24 h, the retained area of span-80/ZNF was 23.63% of the initial area, which was only slightly higher than that of the zein nanofibers without added surfactant (ZNF_200, 20.60%). SEM observations after 24 h of water immersion further revealed morphological changes in nanofiber mats with different fiber diameters (Figure 5) and in those prepared with different surfactants (Figure 6).

### 3.6. DSC Analysis

**Figure 7:**
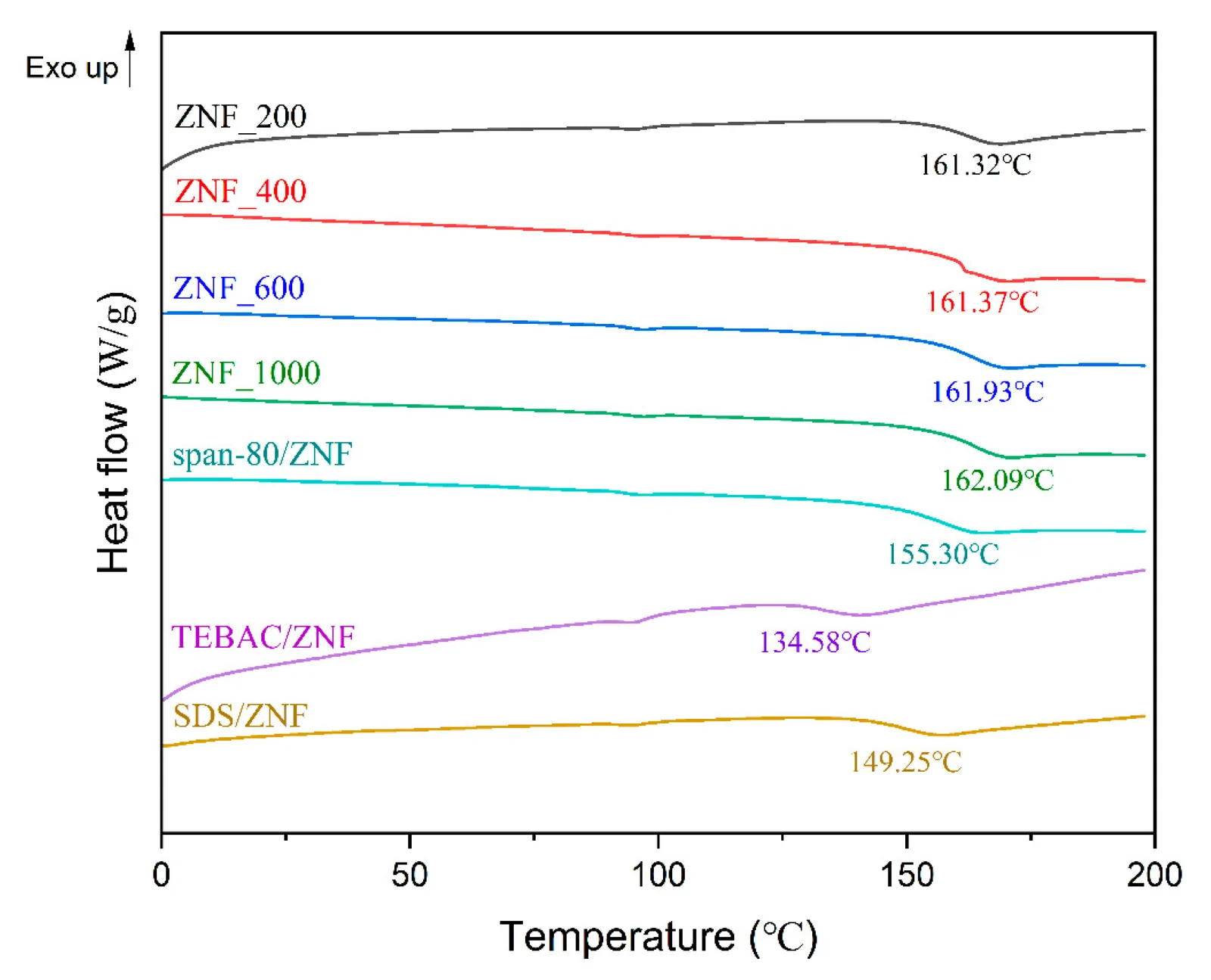
Second-heating of DSC curves and glass transition temperature of zein nanofibers with different diameters and with surfactants added.

As can be seen from Figure 7, zein nanofibers with different diameters exhibited similar thermal behaviors. The glass transition temperatures (T_g_) of ZNF_200, ZNF_400, ZNF_600, and ZNF_1000 were 161.32 °C, 161.37 °C, 161.93 °C, and 162.09 °C, respectively. As the fiber diameter increased, the T_g_ value of the zein nanofibers slightly increases. However, the addition of surfactants leads to a decrease in the T_g_ value of the zein nanofibers. The T_g_ values of TEBAC/ZNF, SDS/ZNF, and span-80/ZNF were 134.58 °C, 149.25 °C, and 155.30 °C, respectively, indicating a reduction in the thermal stability of the zein nanofibers after the addition of surfactants. The different T_g_ values of zein nanofibers assisted by different types of surfactants may be attributed to the varying interactions between surfactants and zein molecules.

### 3.7. Thermal Gravimetric Analysis

**Figure 8:**
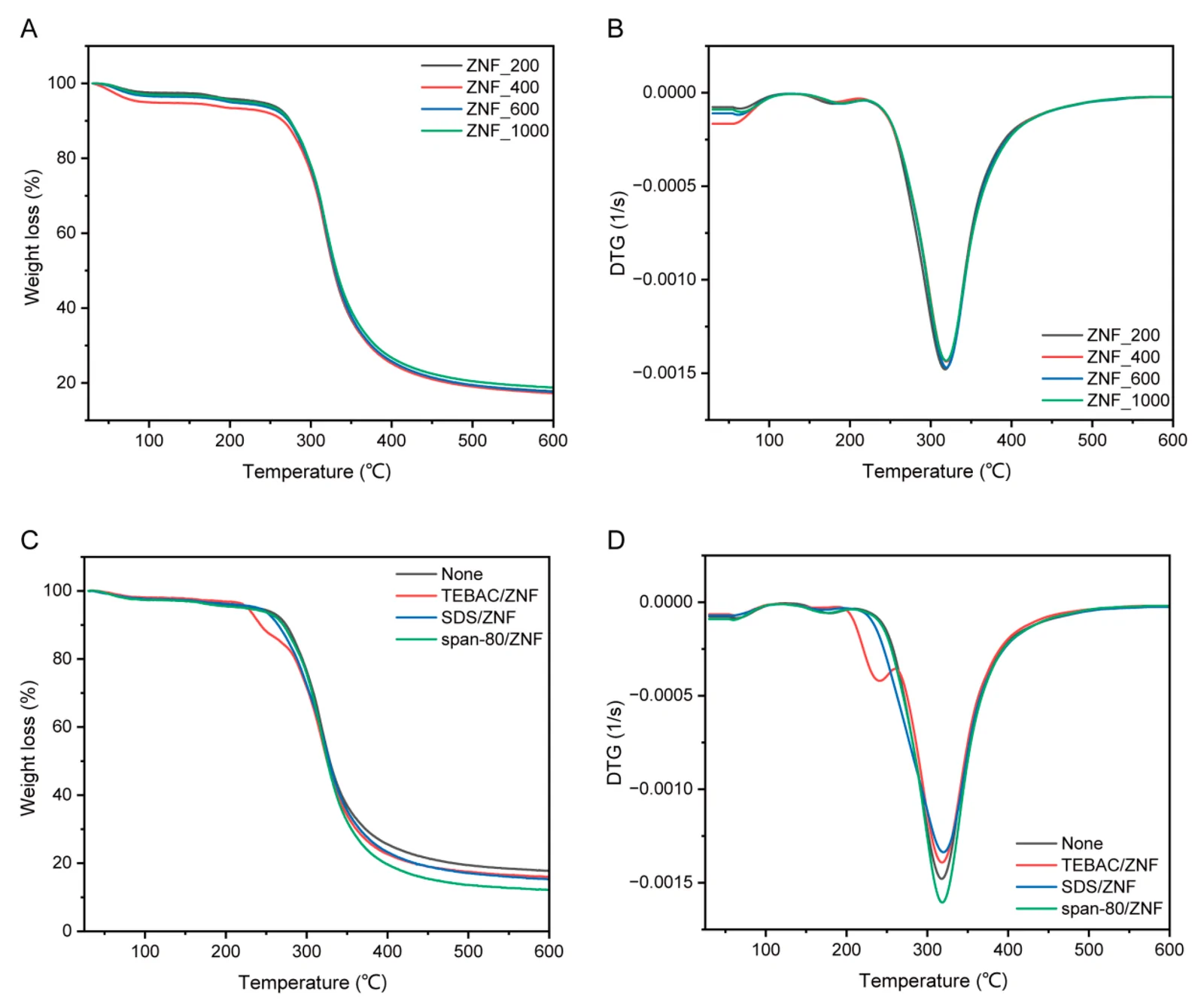
Thermogravimetric curves (**A**,**C**) and DTG curves (**B**,**D**) of zein nanofibers with different diameters (**A**,**B**) and with surfactants added (**C**,**D**).

The thermogravimetric profiles (Figure 8) showed that the weight loss of zein nanofibers could be divided into three stages. The first stage occurred below 100 °C and was mainly attributed to the evaporation of water in the samples; the weight loss during this stage is related to the hydrophilicity and moisture content of the zein nanofiber samples. The weight loss in the second stage occurs within the range of 100–225 °C and is primarily attributed to the thermal decomposition of low molecular weight peptides. The weight loss in the third stage occurs between 225 and 600 °C, during which the thermal decomposition of zein primarily took place. ZNF_200, ZNF_400, and ZNF_600 exhibited similar thermal degradation behaviors, with residual masses of 17.75%, 17.23%, and 17.62%, respectively, at 600 °C. Conversely, ZNF_1000 demonstrated enhanced thermal stability, with a residual mass of 18.76%. However, the residual masses of TEBAC/ZNF, SDS/ZNF, and span-80/ZNF at 600 °C were 16.04%, 15.27%, and 12.16%, respectively, indicating that the presence of surfactants reduced the thermal stability of the zein nanofibers, which is consistent with the DSC results described above. In the study by de Almeida et al. [11], the presence of the cationic surfactant cetylpyridinium bromide (CPB) in cellulose acetate nanofibers led to improved thermal stability. The discrepancy may be attributed to differences in the physicochemical properties of the polymer–solvent systems investigated. Compared with cellulose acetate, zein possesses a more hydrophobic molecular structure, while the polar protic solvent acetic acid used in this study may result in distinct polymer–solvent–surfactant interactions, thereby altering the effects of surfactants on the solution properties.

### 3.8. FTIR Measurements

**Figure 9:**
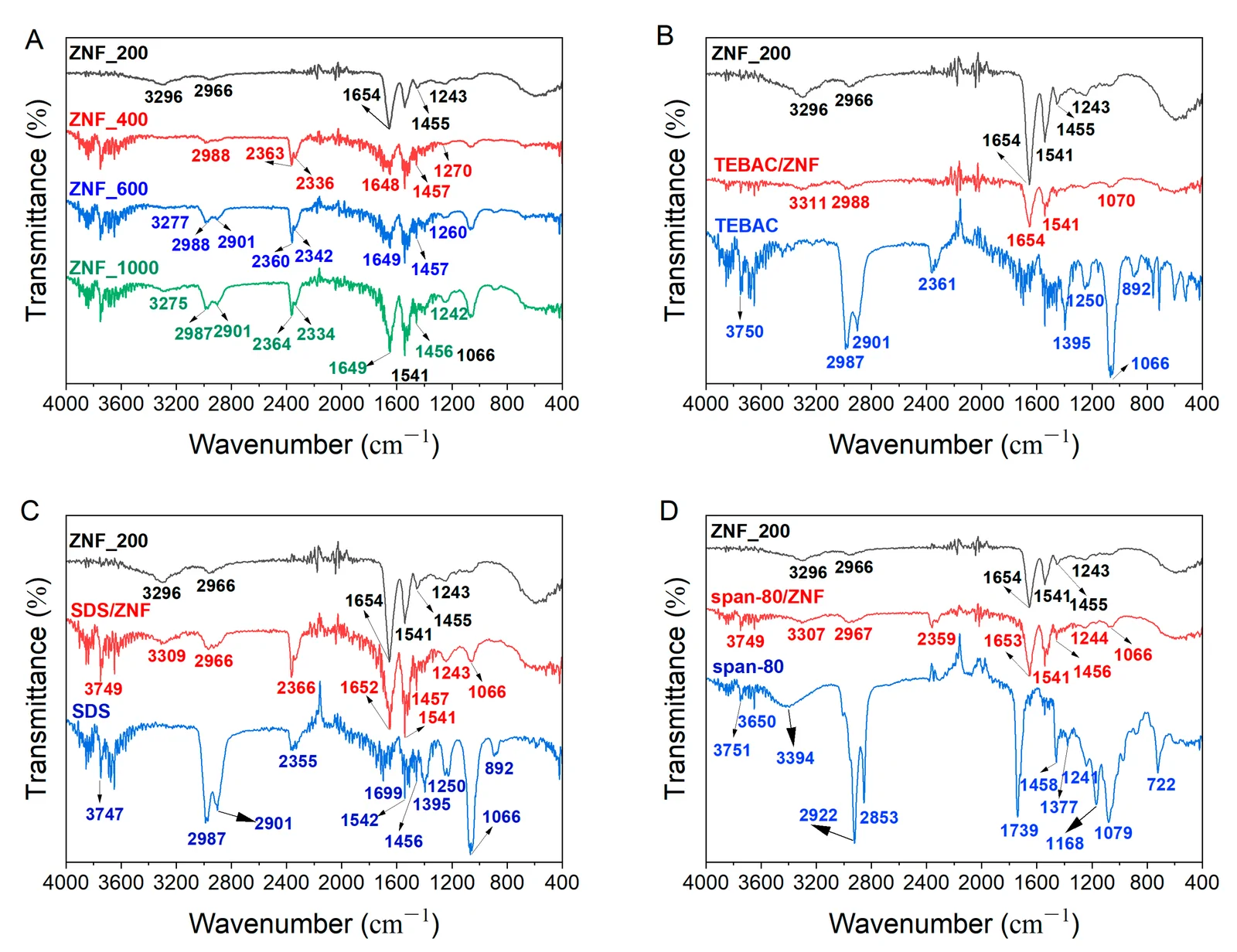
FTIR spectra of (**A**) zein nanofibers with different fiber diameters; (**B**) ZNF_200, TEBAC–modified zein nanofibers (TEBAC/ZNF), and TEBAC; (**C**) ZNF_200, SDS–modified zein nanofibers (SDS/ZNF), and SDS; and (**D**) ZNF_200, span 80–modified zein nanofibers (Span 80/ZNF), and span-80.

In infrared spectroscopy, the characteristic peak located at 3292–3305 cm^−1^ is attributed to N–H stretching vibration and hydrogen bonding [42]. The characteristic peak observed at 2960 cm^−1^ corresponds to the C–H bond in the CH_3_ functional group of zein [22]. The characteristic peak at 1730 cm^−1^ is attributed to the stretching vibration of the uncoupled C=O bond [11]. The characteristic peak at 1654 cm^−1^ corresponds to the stretching vibration of the C=O bond in the amide I band [13]. The characteristic peaks at 1541–1544 cm^−1^ were assigned to the amide II vibration, arising mainly from N–H bending coupled with C–N stretching [42]. The band at approximately 1455 cm^−1^ was attributed to CH_2_/CH_3_ bending vibrations, whereas the bands at 1242–1247 cm^−1^ were assigned to the amide III region, which primarily involves C–N stretching coupled with N–H bending [42,43]. As shown in Figure 9A, ZNF_200, ZNF_400, ZNF_600, and ZNF_1000 exhibited similar spectral characteristics, indicating that fiber diameter does not alter the fundamental structure of zein nanofibers. Furthermore, the peak intensities at 1066 cm^−1^ and 1541 cm^−1^ gradually increased with the increase in fiber diameter. As the fiber diameter increased, the characteristic peaks located at 1243 cm^−1^, 1455 cm^−1^, and 2966 cm^−1^ exhibited a blue shift, while the characteristic peak at 1654 cm^−1^ underwent a red shift. These results indicate that the interactions among zein–zein molecular chains vary in nanofibers with different diameters. During the electrospinning process, a higher polymer concentration leads to greater molecular–chain entanglement under the same stretching force, resulting in stronger interactions among molecular chains.

The infrared spectra of the surfactants, zein nanofibers, and surfactant–assisted zein nanofibers were measured to characterize the interactions between surfactants and zein molecules. As presented in Figure 9B, the peak intensities at 1654 cm^−1^ and 1541 cm^−1^ in the infrared spectrum of TEBAC/ZNF decreased significantly, mainly due to the interaction between TEBAC and zein. Furthermore, the addition of TEBAC caused the characteristic peaks located at 3296 cm^−1^ and 2966 cm^−1^ in the zein nanofibers to shift to 3311 cm^−1^ and 2988 cm^−1^, respectively, indicating the formation of a micellar structure between TEBAC and zein molecules. According to Figure 9C, the incorporation of SDS altered the intensities of several characteristic bands of zein, particularly those near 2966, 1541, and 1243 cm^−1^. The band near 1243 cm^−1^ may also contain contributions from the asymmetric S–O stretching vibration of SDS. In addition, the amide I band shifted slightly from 1654 to 1652 cm^−1^, suggesting changes in the local molecular environment of zein after SDS incorporation, which may be associated with electrostatic interactions between SDS and zein molecules. In Figure 9D, the incorporation of Span 80 altered the intensities of the bands near 2967 and 1541 cm^−1^, while shifts in the bands at 3296, 2966, and 1455 cm^−1^ indicated changes in the molecular environment of zein. These spectral changes suggest interactions between Span 80 and zein, likely dominated by non-electrostatic interactions. The weak high-wavenumber features observed near 3749 cm^−1^ and 2359–2366 cm^−1^ were attributed primarily to residual atmospheric H_2_O and CO_2_ interference, respectively, and were therefore excluded from the interpretation of zein–surfactant interactions [44].

The secondary structure compositions of zein nanofibers with different fiber diameters and those prepared with surfactants are presented in Table 3. As the fiber diameter increased, the contents of α-helix and β-sheet structures increased, whereas the β-turn content decreased significantly. Wang et al. [45] also reported consistent results, showing that increasing protein concentration from 30% to 40% resulted in a 50% increase in the ratio of α-helix/β-structure in the nanofibers. Moreover, the content of β-turn structures in the zein nanofibers decreased to varying degrees after the incorporation of different surfactants, while the content of α–helix structures increased accordingly. Among the surfactant–containing nanofibers, SDS/ZNF showed the most pronounced changes in secondary structure, whereas TEBAC/ZNF showed the least pronounced changes. These results indicate that the behavior of cationic, anionic, and non-ionic surfactants in zein solution is different, depending on the inherent properties and interactions among the surfactant, solvent, and zein molecules [14].

### 3.9. Formation Mechanism of Surfactant-Assisted Zein Nanofibers

For a pure zein solution, the solution is delivered to the capillary tip, where it forms a positively charged pendant droplet. Upon application of the electric field, the droplet is stretched into an elliptical configuration and then extended into a jet. Throughout this process, solvent evaporation and rapid jet solidification occur, culminating in the formation of nanofibers. Within the standard spinnable concentration bracket, decreasing the zein concentration facilitates the formation of fibers with smaller diameters, primarily because of the associated decrease in solution viscosity. As the zein concentration decreases, intermolecular chain entanglement becomes weaker. As a result, under the same electrostatic stretching conditions, fibers with smaller diameters are produced. However, outside the spinnable concentration bracket, an excessively high zein concentration may cause nozzle clogging because of the high solution viscosity, while an excessively low zein concentration may result in electrospraying and nanoparticle generation because of insufficient molecular chain entanglement. Upon the addition of surfactants, interactions may occur among zein chains, surfactant molecules, and solvent molecules, resulting in the formation of molecular associations in the solution. The formation of these molecular associations markedly modifies the characteristics of the polymer solution [33]. When the surfactant is added at a concentration above the critical micelle concentration (CMC), surfactant molecules self-assemble into micelles within the solution. These micellar assemblies move randomly in the solution and may associate with zein molecules or zein aggregates or adsorb onto the surface of zein aggregates. Surfactant adsorption at the gas–liquid interface lowers the surface tension of the zein solution. Additionally, given the relatively high mobility within the electrospun polymer jet, surfactant molecules can migrate to the gas–liquid interface, thereby amplifying the charge density of the droplets and increasing the electrical conductivity.

**Figure 10:**
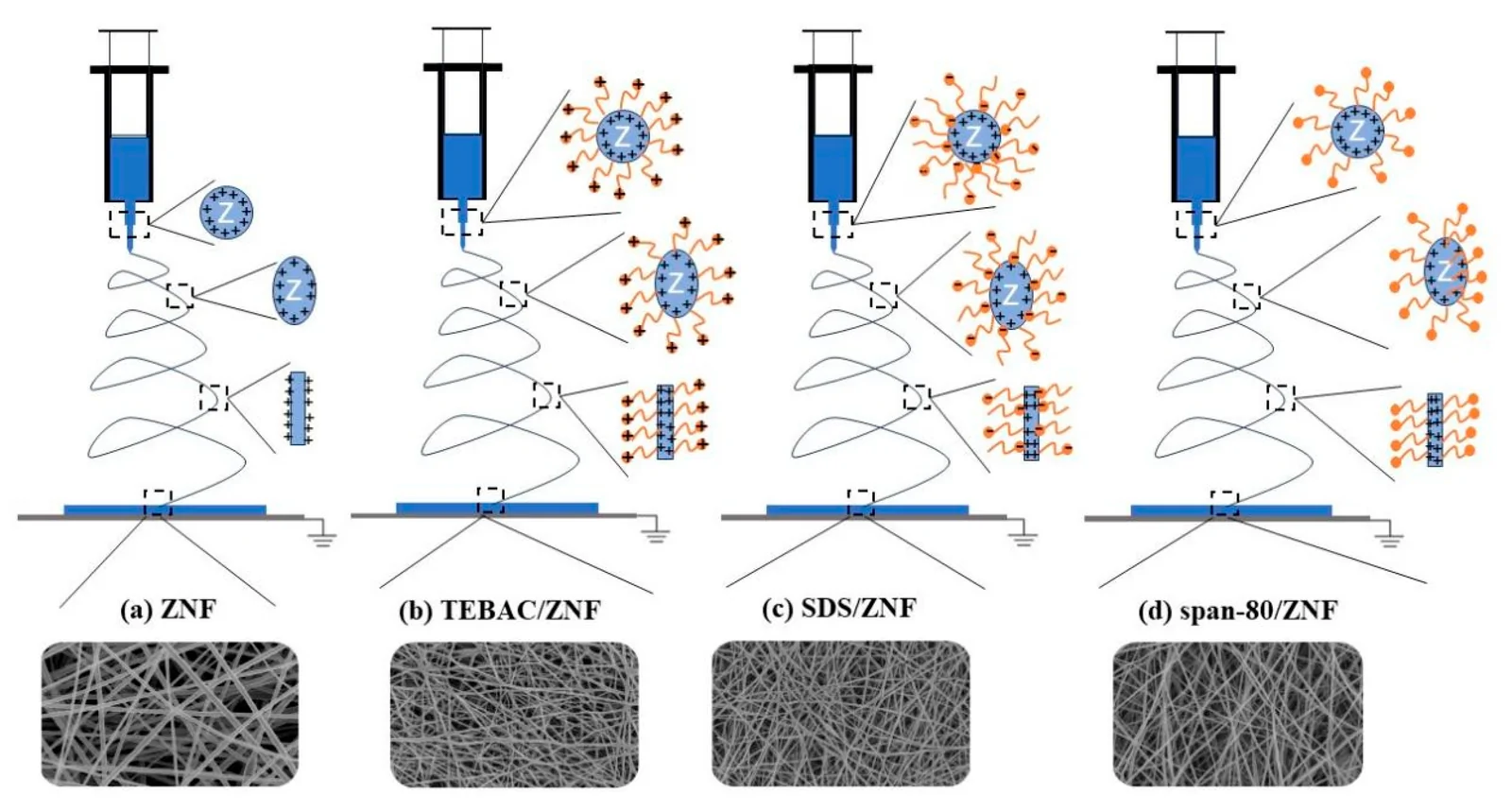
Effect of surfactants on the molecular structure of zein nanofibers.

In glacial acetic acid, zein is positively charged. Owing to the hydrophobic nature of zein and differences in the charge characteristics of the surfactant head groups, different surfactants interact differently with zein in solution. Figure 10 presents the formation process of zein fiber membranes and the distribution of surfactants across the solution and the fibers. The non-ionic surfactant span-80, being charge-free, when added to the zein solution, causes a slight increase in conductivity and a decrease in the surface tension of the mixed solution. TEBAC is a cationic surfactant with a positively charged head group. Its addition significantly increased the conductivity and caused a distinct decline in the surface tension of the mixed solution. However, since the anionic surfactant SDS carries a negative charge, which partially offsets the positive charge on zein, the TEBAC/zein mixed solution demonstrates the highest conductivity and the lowest surface tension relative to the SDS/zein and span-80/ZNF mixed solutions. The combination of reduced surface tension and enhanced conductivity promotes a greater extent of stretching of zein molecular chains during electrospinning, consequently producing fibers with reduced diameters.

## 4. Conclusions

This study regulated the fiber diameter and morphology of electrospun zein fibers by optimizing electrospinning parameters and incorporating cationic surfactant (TEBAC), anionic surfactant (SDS), or nonionic surfactant (span-80). The results showed that optimization of electrospinning parameters reduced zein fiber diameter to a certain extent, whereas surfactant incorporation produced a substantially greater fiber-refining effect and promoted the formation of ultrafine fibers with diameters approaching 100 nm. The different surfactants exerted distinct regulatory effects on fiber formation and material properties because of their specific interactions with zein. Changes in solution conductivity and surface tension influenced jet stretching and fiber refinement, while alterations in intermolecular associations and protein secondary structure further determined the organization and macroscopic performance of the fiber networks. TEBAC facilitated the formation of a stronger and more dimensionally stable fiber network, whereas SDS and span-80 increased flexibility but reduced tensile strength and stiffness. All three surfactants improved fiber wettability, although this improvement was accompanied by decreased thermal stability.

Surfactant-assisted electrospinning can simultaneously regulate the nanoscale morphology, molecular structure, and functional properties of zein fibers. Reducing the fiber diameter to approximately 100 nm substantially increases the surface-area-to-volume ratio and interfacial area of the fibrous network, providing more accessible interaction sites and shorter diffusion pathways for active-compound loading and release, adsorption, filtration, and other interfacial processes. More importantly, achieving zein fibers at this scale demonstrates the feasibility of precisely controlling protein-based fiber formation through coordinated regulation of electrospinning conditions, solution properties, and zein–surfactant interactions. The relationships established among processing conditions, molecular structural changes, fiber diameter, and material performance provide both a mechanistic understanding and a methodological basis for the development of application–oriented protein-based nanofibrous materials. Nevertheless, further studies are needed to assess the scalability and reproducibility of surfactant-assisted electrospinning and to validate the performance of the resulting nanofibrous materials in practical applications, including active food packaging and controlled delivery systems. In particular, their potential use as functional coating layers or carriers of antioxidant and antimicrobial agents for fresh and processed lamb products warrants further investigation to mitigate lipid oxidation and microbial spoilage and thereby extend product shelf life.

## Figures and Tables

**Table 1:** The parameters used for electrospinning.

Samples	Mass Concentration of Zein (%)	Flow Rate (mL/h)	Voltage (kV)	Collection Distance (cm)	Speed (rpm)
ZNF_30_0.5	30	0.5	20	10	1000
ZNF_35_1	35	1.0	20	10	1000
ZNF_40_1	40	1.0	20	10	1000
ZNF_40_3	40	3.0	20	10	1000
TEBAC/ZNF	30	0.5	20	10	1000
SDS/ZNF	30	0.5	20	10	1000
span-80/ZNF	30	0.5	20	10	1000

**Table 2:** The viscosities, electrical conductivity, and surface tension of the feed solutions.

Samples	Viscosity (Pa·s)	Electrical Conductivity (μS/cm)	Surface Tension (mN/m)	Diameter (nm)
ZNF_30_0.5	0.32 ± 0.004 ^cB^	103.73 ± 0.32 ^eD^	46.10 ± 0.05 ^cA^	208.22 ± 46.93 ^dA^
ZNF_35_1	0.64 ± 0.005 ^b^	113.67 ± 0.47 ^d^	47.07 ± 0.04 ^b^	391.40 ± 60.85 ^c^
ZNF_40_1	1.06 ± 0.054 ^a^	102.83 ± 0.65 ^e^	47.98 ± 0.06 ^a^	602.28 ± 82.52 ^b^
ZNF_40_3	1.06 ± 0.054 ^a^	102.83 ± 0.65 ^e^	47.98 ± 0.06 ^a^	1001.90 ± 121.33 ^a^
TEBAC/ZNF	0.34 ± 0.002 ^cB^	225.67 ± 0.58 ^aA^	36.72 ± 0.13 ^fD^	106.10 ± 18.58 ^eB^
SDS/ZNF	0.38 ± 0.013 ^cA^	154.37 ± 0.65 ^bB^	38.11 ± 0.07 ^eC^	98.92 ± 15.20 ^eC^
span-80/ZNF	0.33 ± 0.022 ^cB^	120.23 ± 0.72 ^cC^	41.08 ± 0.02 ^dB^	106.11 ± 17.98 ^eB^

Results in each column with different lowercase letters indicate significant differences (*p* < 0.05) in all samples. Values in each column with various capital letters indicate significant differences in the properties of the solution with the addition of various surfactants.

**Table 3:** Secondary structure content of zein nanofibers with different diameters and with surfactants added.

Samples	β–Sheets	α–Helix	β–Turns
ZNF_200	42.87 ± 0.69 ^bAB^	33.55 ± 0.35 ^bB^	23.58 ± 0.45 ^aA^
ZNF_400	44.45 ± 0.84 ^a^	34.34 ± 0.35 ^a^	18.74 ± 0.41 ^b^
ZNF_600	45.20 ± 1.02 ^a^	34.02 ± 0.17 ^ab^	18.04 ± 0.31 ^c^
ZNF_1000	45.67 ± 0.73 ^a^	34.04 ± 0.26 ^ab^	17.48 ± 0.28 ^c^
TEBAC/ZNF	42.45 ± 0.67 ^B^	33.04 ± 0.44 ^C^	22.34 ± 0.51 ^B^
SDS/ZNF	43.71 ± 0.59 ^A^	34.16 ± 0.65 ^A^	19.89 ± 0.88 ^C^
span-80/ZNF	42.16 ± 0.47 ^B^	34.37 ± 0.51 ^A^	21.74 ± 0.42 ^B^

Results in each column with different lowercase letters indicate significant differences (*p* < 0.05) in zein nanofibers with different diameters. Results in each column with different capital letters indicate significant differences (*p* < 0.05) in zein nanofibers with various surfactants added.

## Data Availability

The original contributions presented in the study are included in the article/Supplementary Materials; further inquiries can be directed to the corresponding authors.

## Supplementary Materials

The following supporting information can be downloaded at: https://www.mdpi.com/article/10.3390/foods15173156/s1, Figure S1: The images of zein nanofibers with different diameters and with surfactants added after immersion in water for 24 h; Figure S2: Fiber diameter distributions of electrospun zein nanofibers with different average diameters and surfactant-containing zein nanofibers. The average fiber diameter is indicated in each panel; Table S1: Physical properties of zein solutions with various surfactant and concentrations added.

## References

[1] Al-Hammood O., Oladzadabbasabadi N., Mohammed A.H., Al-Musawi M.H., PourvatanDoust S., Ghorbani M. (2025). Electrospun-Modified Xanthan Gum Nanofibers Enhanced with Nisin for Food Packaging Applications. Int. J. Biol. Macromol..

[2] Lu Z., Qin M., Wang Z., Yang H., Han Q., Sun J., Sha J., Zhu Y., Bai Z. (2025). Correlating Cone/Jet Features of Electrospinning with Fiber Diameter and Wettability to Modulate Fiber Membranes with Different Wettabilitiy. Chem. Eng. Sci..

[3] Almafie M.R., Fudholi A., Dani R., Idjan M.K.N.A., Royani I., Sriyanti I. (2025). Effects of Electrospinning Parameters on Polycaprolactone Membrane Diameter: An Investigation Utilizing Central Composite Design and Characterization. Results Eng..

[4] Chen Y.-J., Chang T.-L., Wu Q.-X., Ke K.-C., Chiu P.-S., Chen C.-T. (2025). Investigation and Predictive Multi-Modeling of Pva/Pvp-Blended Nanofiber Diameter in Electrospinning. Int. J. Adv. Manuf. Technol..

[5] Shen C., Yang Z., Wu D., Chen K. (2024). The Preparation, Resources, Applications, and Future Trends of Nanofibers in Active Food Packaging: A Review. Crit. Rev. Food Sci. Nutr..

[6] Nasrabadi M.N., Doost A.S., Mezzenga R. (2021). Modification Approaches of Plant-Based Proteins to Improve Their Techno-Functionality and Use in Food Products. Food Hydrocoll..

[7] Mattice K.D., Marangoni A.G. (2020). Comparing Methods to Produce Fibrous Material from Zein. Food Res. Int..

[8] Patel D.K., Won S.-Y., Jung E., Han S.S. (2025). Recent Progress in Biopolymer-Based Electrospun Nanofibers and Their Potential Biomedical Applications: A Review. Int. J. Biol. Macromol..

[9] Arif A.R., Shafar Z., Rizki W.O.S., Mozef T., Ivansyah A.L., Nasir M., Hertadi R. (2026). Revealing the Effects of Process Parameters on Structural, Thermal, Mechanical, Biodegradation, and Biocompatibility Properties on the Electrospinning of Poly (Vinyl Alcohol)/Microbial Inulin Nanofibers. ACS Omega.

[10] Mhetre H.V., Krishnarao K.Y., Naik N. (2023). Optimization of Electrospinning Process Parameters to Develop the Smallest Zno+ Pvp Nanofibres Using Taguchi Experimental Design and Anova. J. Mater. Sci. Mater. Electron..

[11] de Almeida D.S., Duarte E.H., Hashimoto E.M., Turbiani F.R., Muniz E.C., de Souza P.R., Gimenes M.L., Martins L.D. (2020). Development and Characterization of Electrospun Cellulose Acetate Nanofibers Modified by Cationic Surfactant. Polym. Test..

[12] Wang S.Q., He J.H., Xu L. (2008). Non-Ionic Surfactants for Enhancing Electrospinability and for the Preparation of Electrospun Nanofibers. Polym. Int..

[13] Shao P., Liu Y., Ritzoulis C., Niu B. (2019). Preparation of Zein Nanofibers with Cinnamaldehyde Encapsulated in Surfactants at Critical Micelle Concentration for Active Food Packaging. Food Packag. Shelf Life.

[14] Beigmoradi R., Samimi A., Mohebbi-Kalhori D. (2021). Controllability of the Hydrophilic or Hydrophobic Behavior of the Modified Polysulfone Electrospun Nanofiber Mats. Polym. Test..

[15] Aytac Z., Huang R., Vaze N., Xu T., Eitzer B.D., Krol W., MacQueen L.A., Chang H., Bousfield D.W., Chan-Park M.B. (2020). Development of Biodegradable and Antimicrobial Electrospun Zein Fibers for Food Packaging. ACS Sustain. Chem. Eng..

[16] Xia L., Li L., Xiao Y., Xiao F., Ji W., Pang M., Wang H. (2023). Hydrophobic Electrospun Membrane of Peppermint Oil Loaded Zein with Coating of Methyltriethoxysilane for Active Packaging. Food Hydrocoll..

[17] Wang S., Wang P., Liu S., Wang R., Li Y., Wang X., Ren F., Luo J., Fang B. (2024). Enhancement of Mechanical Properties of Zein-Based Nanofibers by Incorporation of Millet Gliadin. Foods.

[18] Rahati S., Hamishehkar H., Torbati M., Mohammadi M., Tabibiazar M., Azadmard-Damirchi S. (2025). Electrospinning: An Emerging Technology for Enhancing the Quality and Ensuring the Safety of Seafood Products–A Comprehensive Review. Trends Food Sci. Technol..

[19] Selling G.W., Biswas A., Patel A., Walls D.J., Dunlap C., Wei Y. (2007). Impact of Solvent on Electrospinning of Zein and Analysis of Resulting Fibers. Macromol. Chem. Phys..

[20] da Silva N.M.A., Silva L.P. (2026). A Comprehensive Review on Food-Grade Electrospinning of Natural Biopolymers for Cultivated Meat Applications. Foods.

[21] Khan M.A., Chen L., Liang L. (2021). Improvement in Storage Stability and Resveratrol Retention by Fabrication of Hollow Zein-Chitosan Composite Particles. Food Hydrocoll..

[22] Barakat H.S., Freag M.S., Gaber S.M., Al Oufy A., Abdallah O.Y. (2023). Development of Verapamil Hydrochloride-Loaded Biopolymer-Based Composite Electrospun Nanofibrous Mats: In Vivo Evaluation of Enhanced Burn Wound Healing without Scar Formation. Drug Des. Dev. Ther..

[23] Yan X., Li M., Xu X., Liu X., Liu F. (2022). Zein-Based Nano-Delivery Systems for Encapsulation and Protection of Hydrophobic Bioactives: A Review. Front. Nutr..

[24] Charpashlo E., Ghorani B., Mohebbi M. (2021). Multilayered Electrospinning Strategy for Increasing the Bioaccessibility of Lycopene in Gelatin-Based Sub-Micron Fiber Structures. Food Hydrocoll..

[25] Khan K.-u.-H., Siddiqui I.A. (2025). A Predictive Model for Electrospun Based Polyvinyl Alcohol (Pva) Nanofibers Diameter Using an Artificial Neural Network. Sci. Rep..

[26] Liu Y., Wang Y., Lee C.-H., Kan C.-W., Lu X. (2024). Influence of Electrospinning Parameters on the Morphology of Electrospun Poly (3-Hydroxybutyrate-Co-3-Hydroxyvalerate) Fibrous Membranes and Their Application as Potential Air Filtration Materials. Polymers.

[27] Yusro M., Hacker V. (2024). Fabricating Ultra-Thin Nanofiber Structures Towards the Advanced Mea of Fuel Cells: Investigation of the Degree of Alignment, Diameter, Bead Generation, and Precision with Taguchi Designs. Nanoscale Adv..

[28] Sorkhabi T.S., Samberan M.F., Ostrowski K.A., Zajdel P., Stempkowska A., Gawenda T. (2022). Electrospinning of Poly (Acrylamide), Poly (Acrylic Acid) and Poly (Vinyl Alcohol) Nanofibers: Characterization and Optimization Study on the Effect of Different Parameters on Mean Diameter Using Taguchi Design of Experiment Method. Materials.

[29] Wang S., Li J., Wang P., Zhang M., Liu S., Wang R., Li Y., Ren F., Fang B. (2024). Improvement in the Sustained-Release Performance of Electrospun Zein Nanofibers Via Crosslinking Using Glutaraldehyde Vapors. Foods.

[30] Leena M.M., Yoha K., Moses J., Anandharamakrishnan C. (2020). Edible Coating with Resveratrol Loaded Electrospun Zein Nanofibers with Enhanced Bioaccessibility. Food Biosci..

[31] Isik B.S., Bayram S., Altay F. (2025). Electrospinning Encapsulation of Chlorophylls Microwave-Extracted from Spinach Waste and Utilization as Coloring Agent in Yogurt. Food Bioprocess Technol..

[32] Guo J., Chen B., Shen Y., Chen C., Yang Y., Yu D. (2025). Morphological Modulation of Electrospun Pvdf/Pvp Nanofibers. J. Polym. Sci..

[33] Shan X., Li F., Liu C., Gao Q. (2014). Electrospinning of Chitosan/Poly (Lactic Acid) Nanofibers: The Favorable Effect of Nonionic Surfactant. J. Appl. Polym. Sci..

[34] Ziani K., Henrist C., Jérôme C., Aqil A., Maté J.I., Cloots R. (2011). Effect of Nonionic Surfactant and Acidity on Chitosan Nanofibers with Different Molecular Weights. Carbohydr. Polym..

[35] Jia L., Qin X.-h. (2013). The Effect of Different Surfactants on the Electrospinning Poly (Vinyl Alcohol)(Pva) Nanofibers. J. Therm. Anal. Calorim..

[36] Haghi A., Akbari M. (2007). Trends in Electrospinning of Natural Nanofibers. Phys. Stat. Sol. (a).

[37] Lu H., Wang Q., Li G., Qiu Y., Wei Q. (2017). Electrospun Water-Stable Zein/Ethyl Cellulose Composite Nanofiber and Its Drug Release Properties. Mater. Sci. Eng. C.

[38] Ghorani B., Tucker N. (2015). Fundamentals of Electrospinning as a Novel Delivery Vehicle for Bioactive Compounds in Food Nanotechnology. Food Hydrocoll..

[39] Sari B., Kaynak C. (2024). Parameters Influencing Electrospun Nanofiber Diameter of Polylactide Incorporated with Cellulose Nanofibrils and Nanocrystals. J. Thermoplast. Compos. Mater..

[40] Liu W., Walker G., Price S., Yang X., Li J., Bunt C. (2021). Electrospun Membranes as a Porous Barrier for Molecular Transport: Membrane Characterization and Release Assessment. Pharmaceutics.

[41] Wenzel R.N. (1936). Resistance of Solid Surfaces to Wetting by Water. Ind. Eng. Chem..

[42] Deng L., Kang X., Liu Y., Feng F., Zhang H. (2017). Effects of Surfactants on the Formation of Gelatin Nanofibres for Controlled Release of Curcumin. Food Chem..

[43] Noman R.R.A., Wong C.S., Law K.P., Neo Y.P. (2024). Fabrication and Characterisation of Electrospun Zein-Based Fibres Functionalised by Caffeic and P-Coumaric Acid for Potential Active Packaging Applications. Int. J. Food Sci. Technol..

[44] Bruun S.W., Kohler A., Adt I., Sockalingum G.D., Manfait M., Martens H. (2006). Correcting Attenuated Total Reflection–Fourier Transform Infrared Spectra for Water Vapor and Carbon Dioxide. Appl. Spectrosc..

[45] Wang Y., Zhao M., Barker S.A., Belton P., Craig D. (2019). A Spectroscopic and Thermal Investigation into the Relationship between Composition, Secondary Structure and Physical Characteristics of Electrospun Zein Nanofibers. Mater. Sci. Eng. C.

