# Supplementary material for: Formation and Properties of Electrospun Zein Nanofibers from Glacial Acetic Acid Solutions: Effects of Process Variables and Surfactants
Source: Foods. 2026 Sep 5;15(17):3156. doi: 10.3390/foods15173156 (PMC13565642; doi:10.3390/foods15173156)
Supplement: Supplementary file 1 [file foods-15-03156-s001.zip › foods-4509320-supplementary.pdf]

Table S1. Physical properties of zein solutions with various surfactant and concentrations added.

Samples	Viscosity (Pa.s)	Surface tension (mN/m)	Electrical conductivity (μS/cm)
None	0.32±0.01^{deC}	46.10±0.05^{aA}	103.73±0.32^{jD}
2%TEBAC	0.34±0.01^{bcdB}	36.72±0.13^{mD}	225.67±0.58^{cC}
10%TEBAC	0.33±0.01^{bcdBC}	37.17±0.02l^{C}	400.50±2.12^{bB}
20%TEBAC	0.36±0.01^{abA}	37.86±0.03^{kB}	662.00±1.41^{aA}
None	0.32±0.01^{C}	46.10±0.05^{A}	103.73±0.32^{D}
2%SDS	0.38±0.01^{aA}	38.11±0.07^{jD}	154.37±0.65^{eB}
10%SDS	0.34±0.02^{bcdBC}	39.51±0.07^{iC}	142.57±0.57^{fC}
20%SDS	0.36±0.01^{abcAB}	40.06±0.03^{hB}	200.83±0.86^{dA}
None	0.32±0.01^{A}	46.10±0.05^{A}	103.73±0.32^{B}
2%span-80	0.33±0.02^{cdA}	41.08±0.02^{gD}	120.23±0.72^{iA}
10%span-80	0.26±0.01^{gB}	41.76±0.16^{fC}	90.73±0.57^{kC}
20%span-80	0.28±0.03^{fgAB}	42.31±0.08^{eB}	63.73±0.35^{lD}

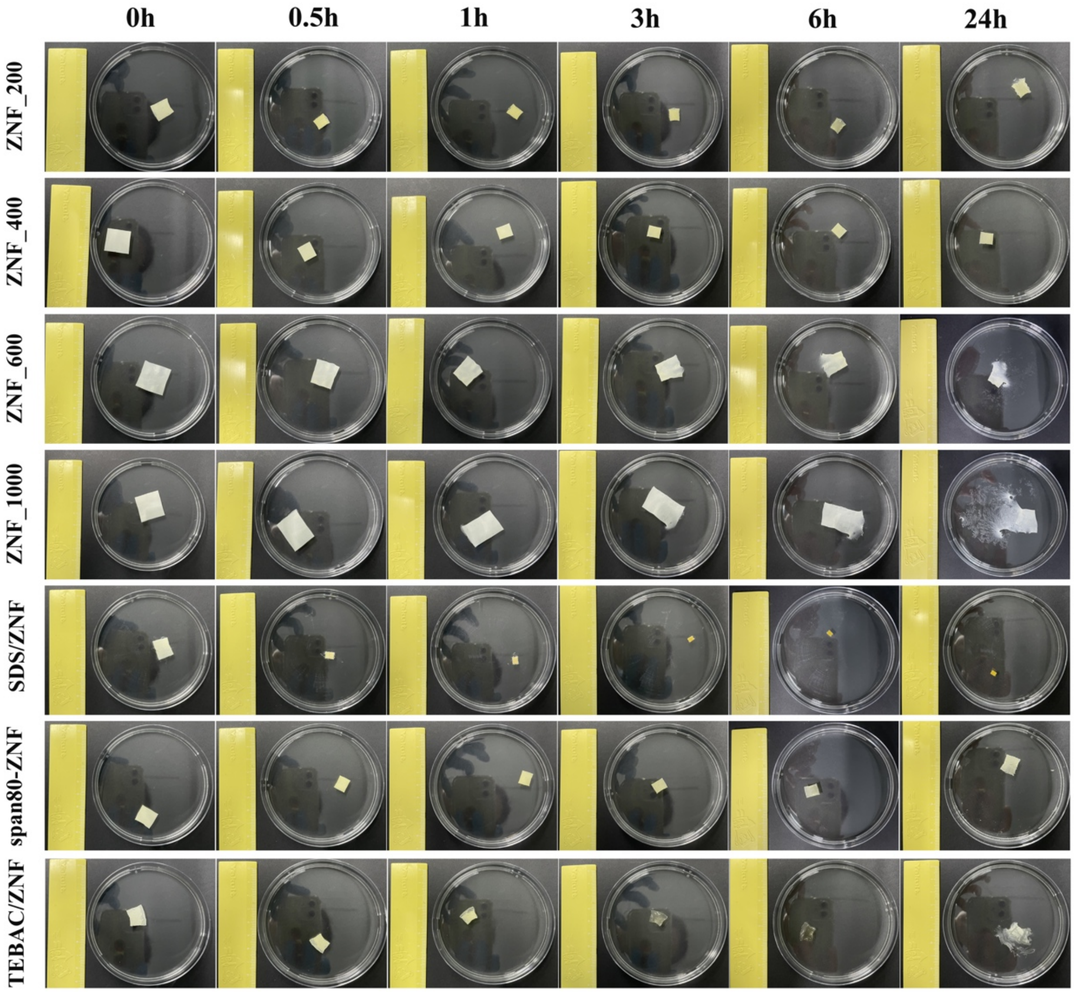

Figure S1. The images of zein nanofibers with different diameters and with surfactants added after immersion in water for 24 h.

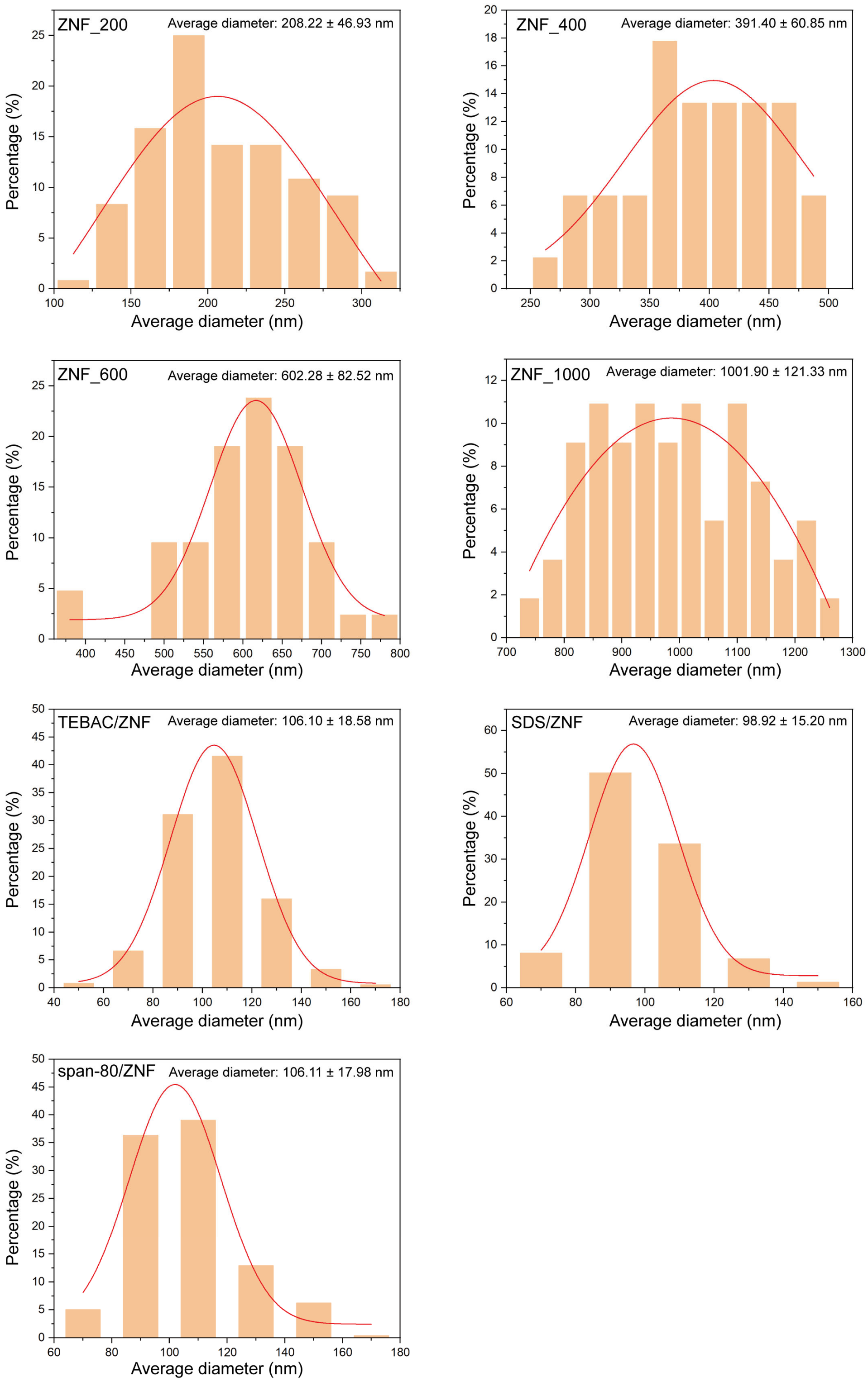

Figure S2. Fiber diameter distributions of electrospun zein nanofibers with different average diameters and surfactant-containing zein nanofibers. The average fiber diameter is indicated in each panel.
